# Successful implementation of change in postgraduate medical education – a qualitative study of programme directors

**DOI:** 10.1186/s12909-021-02606-x

**Published:** 2021-04-14

**Authors:** Hanna Wijk, Kristiina Heikkilä, Sari Ponzer, Lars Kihlström, Jonas Nordquist

**Affiliations:** 1 Department of Medicine (Huddinge), Karolinska Institutet, 141 86 Stockholm, Stockholm, Sweden; 2 Department of Health and Care Sciences, Linnaeus University, Kalmar, Sweden; 3 Department of Clinical Science and Education, Karolinska Institutet, Södersjukhuset, Stockholm, Sweden; 4 Department of Research and Education, Karolinska University Hospital, Stockholm, Sweden

**Keywords:** Qualitative research, Medical education, Leadership, Faculty development, Postgraduate, Change management

## Abstract

**Introduction:**

Leaders in postgraduate medical education are responsible for implementing educational change. Although difficulties in implementing change are described both in the general leadership literature as well as in the field of medical education, knowledge of what characterises successful change leadership in postgraduate medical education is limited. The aim of this study is to explore the process used by educational leaders in successful change implementation in postgraduate medical education.

**Methods:**

Semi-structured interviews were conducted with 16 programme directors to explore how they had implemented successful change projects. The sample consisted of programme directors who had reported in a previous survey having high educational impact at their workplace. Interviews were analysed using Ödman’s qualitative interpretative method.

**Results:**

The interviews identified similarities in how participating programme directors had implemented changes. Five interconnected themes crystallised from the data: (1) belonging to a group, (2) having a vision and meaning, (3) having a mandate for change, (4) involving colleagues and superiors, and (5) having a long-term perspective.

**Conclusions:**

Our findings illuminate important aspects of successful change management in postgraduate medical education. Change is ideally based on a clear vision and is implemented in coalition with others. A long-term strategy should be planned, including involvement and anchoring of key persons in several discrete steps as change is implemented. While some of these findings are congruent with the general literature on change management, this study emphasises the importance of a mandate, with successful change leadership dependent on coalition and the facilitation provided by the next level of leadership.

## Introduction

Educational change is necessary in postgraduate medical education (PGME) due to changes in societal needs, reform in health systems and technological advances. [1–3] In addition, PGME has undergone a change from a time- and process-based system to a competency-based framework. [4, 5].

The implementation of change in medical education, which takes place in the complex and time-constrained environment of healthcare systems, is complicated. [6, 7] It has been concluded that medical educators must increase the focus on how to implement changes [6] and that the use of established theories of change leadership could further guide the change implementation process in PGME. [8] Changes related to the implementation competency-based medical education have been studied and lessons learned include the importance of organisational support and communication among the stakeholders involved in the process. [9–11].

One widely used theory for organisational change leadership is the stepwise model described by John Kotter. [12] Kotter describes an eight-step model which emphasizes the need to engage and involve others. While Kotter’s model has been criticised for having an hierarchical approach to change and being time consuming, it has been shown to be valid for implementing change in many organisations. [13] The model has also been applied to change management in PGME. [14, 15].

In Edmundson’s work [16] on how organizations learn, she highlights the importance of framing and setting a clear vision which enables one to create clear goals. Framing makes it possible to communicate the need for change and the rationale behind a specific initiative. Edmundson´s also emphasizes how important the overall organisational environment is to establish psychological safety, which will increase the likelihood that a change will be successful. [16, 17].

Leadership behaviours in relation to successful change is another track in the large body of research on change. [18] Higgs and Rowland [19] found that leader-centric behaviours had a negative impact on the success of change in all contexts examined; in contrast group- and systemic-focused behaviours were positively related to success in most contexts. Another widely used model is the task-relationship model. Examples of task-oriented behaviours related to change are activities linked to step-by-step-plans, procedures and roles whilst person-oriented behaviours are related to communication, motivation and facilitation. [18, 20].

Educational leaders in PGME, which are referred to as programme directors (PDs) for the purposes of this study, should ensure implementation of changes. [21, 22] However, PDs have limited awareness of change strategies [23] and medical leaders both at under- and the postgraduate levels describe challenges in engaging their colleagues in the process of changing medical education. [24–27].

Despite the need for change in PGME and knowledge regarding difficulties in this area, the number of studies that have provided insight on change leadership for PDs at postgraduate level are limited. The aim of this study was to explore the process of successful change implementation for educational leaders in PGME.

## Methods

### Setting

The current study was performed in Sweden. In Sweden, physicians’ postgraduate training can be delivered by all healthcare providers with no university involvement. PGME is regulated by the National Board of Health and Welfare and according to the regulations, the role of PD is a mandatory function at the departments/healthcare centres with residents. [28] The PD role is regulated as a supporting role, while the formal responsibility for the quality of PGME is placed with the head of the department. However, from a practical perspective it is the PD that is responsible for the programme. PDs usually work as consultants in the teaching setting in parallel with their educational assignment.

### Design

We chose a qualitative interpretative research method for this study. [29–31]. Individual interviews were conducted with PDs asking them to describe their experience with implementing change in PGME within the organisation in a semi-structured manner, in order to catch their narratives. The analysis was conducted using an interpretative method [32], based on Ricoeur’s hermeneutical philosophy. [33] This approach was chosen to uncover the underlying meaning of the PD experiences with successful change implementation and included an iterative process in hermeneutical spiral. [32].

### Ethical considerations

Before the interview, participants received written information explaining the study purpose, confidentiality and the right to withdraw from the study at any stage. Informed consent was obtained from all participants before starting the telephone interview. An application for ethical approval for this study was submitted to the regional Ethical Review Board in Stockholm (Reg. no. 2012/1662-31/5). The board decided that formal approval was not required under Swedish law.

### Participants and procedure

The participants of this study were selected from amongst PDs who had participated in an earlier study [27] and who had reported having a high impact on PGME in their workplace. A purposeful sampling procedure was used to find both male and female participants in different specialties and from various geographic locations. Based on these selection criteria, emails were sent out with information about the study and a request to participate to 37 persons. Eight of these no longer served as PDs and seven declined to participate. Six PDs asked to be included but there were difficulties encountered in finding time for the interview. The final cohort consisted of 16 PDs (Table 1). Participants were provided with information about the study and were asked to prepare for the interview by thinking of examples of successful changes in PGME in which they had been involved as a PD.

**Table 1:** Characteristics of participants

Gender	Female: 11 Male: 5
Specialties	Surgery: 3 Medical: 3 General practitioner: 4 Diagnostic and laboratory: 4 Psychiatric: 2
Experience as a PD	< 5 years: 4 5–10 years: 7 > 10 years: 5

### Data collection

Data was collected between August 2018 and January 2019. A semi-structured interview guide was developed, exploring participants’ perceptions of managing change in PGME. All 16 PDs were interviewed individually, by telephone and by a psychologist (HW). After gathering background information, the interviews began with the opening question ‘Can you tell me about any change in PGME with which you have been involved as a PD that you think had a positive result for the residents?’. A series of subsequent questions focused on descriptions about how the participant had implemented the change. In some interviews more than one change process was described. The interviews were ended with a concluding question about what interviewees thought was important for a PD to do to ensure successful change implementation. The interviews lasted between 41 and 66 min (average of 51 min). All interviews were digitally recorded and transcribed verbatim.

### Analysis

The interviews were analysed using Ödman’s interpretative approach [32] to allow for a comprehensive understanding of the meaning the participants gave to their experience by being as open as possible for the underlying meaning and for a new understanding of the experiences. To illuminate the unvoiced meaning – the “surplus of meaning” – Ricoeur [33] suggests a distancing, questioning, and critical approach. Therefore, the analysis started by the first author reading the transcripts repeatedly in order to catch the ideas and the assumptions of the meaning of the interview data. A summary, including a preliminary interpretation of the meaning of each interview text was created, resulting in a preliminary set of themes with descriptions (Table 2). In the next phase, questions were posed to the interview text to test and challenge the preliminary understandings. During the analysis process, sub-themes were created and/or reduced by merging them, thus allowing the analysis to reach internal homogeneity and external heterogeneity. During the analysis process, notes and memos were kept to capture how the process developed. The questioning and challenging the emerging themes were then continued in an iterative process, thus following the thematic analytical model [32] by going back and forth between the researchers’ assumptions, ideas, questions and explanations and a validation of these themes through comparison with the interview texts. Finally, the material was again read from each participant’s own perspective in order to uncover the meanings and assign meanings in a constantly ongoing dialectical act with the original text. [32] The data analysis proceeded until a coherent understanding of the material was achieved.

The analysis was continuously discussed and re-evaluated by three of the authors (HW, JN and KH) to enhance the reliability of the analysis through exploration of different aspects, contradictory information and interpretations. Confirmability of the results was strengthened by supporting the themes and sub-themes with illustrative quotations throughout the results section and by presenting the analysis in a coherent way.

**Table 2:** Examples of themes and descriptions used in the analysis process

Theme	Description	Quote (example)
Having a vision and meaning	A conviction that the changes that the PDs were implementing were important and should have a positive impact on the educational quality.	My argument was that we make it good for residents, then we’ll have the best basis for recruitment. So the rumour will go, “yes it is good working at the paediatric clinic”.
Involving colleagues and superiors	A deliberate and repeated attempt to engage people at different levels within the organisation.	We anchored the plans at all clinics. We went out and talked to everyone; management, supervisors and residents. Everyone had to come up with their input.

### Trustworthiness

Various aspects of trustworthiness were addressed in the research process. Credibility was strengthened through a continual focus on the research question and through the research team seeking consensus on themes during the analysis process. The authors’ different backgrounds allowed reflection of the research seen from different perspectives and throughout the process of analysis, the research team worked to ensure interpretations were based on the data. Transferability was strengthened through purposeful sampling, which ensured a diverse, representative group of participants and by member checking [34] the draft result at a workshop with PDs not participating in the project in order to check the authenticity of the work. The open dialogue between the researchers and the member checking with PDs added to the dependability of the research findings. [29, 35]

## Results

The findings were sorted into five main themes, each with two sub-themes: (1) Belonging to a group; (2) Having a vision and meaning; (3) Having a mandate for change; (4) Involving colleagues and superiors; and (5) Having a long-term perspective (Table 3). It was observed that for most participants, a stepwise strategy of the entire change process wasn’t pronounced. Instead, single activities (mostly at the beginning of the process), own behaviour and contextual influencing factors were described.

### Characteristics of change projects

Each participant described one or several planned organisational changes that had been implemented. All changes were related to one specific specialty program, but the scope varied between a specialty program within one educational unit (clinical department) and several educational units (clinical departments/healthcare centres). The change projects described by participants addressed various aspects of PGME, such as introducing new structures for theoretical courses, reorganising the clinical training programme, and introducing structured workplace-based assessments. Developmental changes (i.e. changes to improve current procedures) dominated, but some of the changes had transformational characteristics (i.e. profound and complex changes) related to workplace-based assessment and the introduction of a competency based medical education. The changes were initiated by the PDs themselves (bottom-up driven) but founded in the regulations for the specialist training.

**Table 3:** Themes and related sub-themes

Theme	Sub-theme
Belonging to a group	- Working as a team - Representing a group
Having a vision and meaning	- Having an inner motivator - Creating commitment in the organisation
Having a mandate for change	- Experiencing trust and power from superiors - Referring to the PD’s own expert knowledge
Involving colleagues and superiors	- Anchoring - Facilitation
Having a long-term perspective	- Being patient - Implementing changes using a step-by-step process

### Belonging to a group

PDs referred to their respective change initiative as a joint project carried out along with others. Half of the participants worked in a formal group with other PDs, others were part of groups that included a manager or residents. If the PD did not belong to an existing group, s/he created a group for support and to discuss their experience.

“Of course, we were at the same clinic but we had never really worked together and it worked really well. It was great fun and we had a lot of ideas that we bounced off each other and worked through. (…) It was an amazing development to be able to work together in a team then.” (IP 1).

The theme of group membership encompassed two sub-themes. Working as a team included participants’ experience of carrying out the various tasks associated with the change project in collaboration with others. This was manifest in the use of “we” instead of “I” in the interviews and by describing cooperation in a group in parallel with the change process, including discussion of how the tasks were divided amongst the group.

“There are three of us who work together a lot and generally agree with each other. So, when we hit on a lot of different things and then we reach the point where: this [change project] is actually at the heart of the needs.” (IP 9).

The second sub-theme was related to the PDs’ internal feeling of increased power when they represented a group. The PD was not alone in being responsible and driving the change and gained power and strength by belonging to and representing a larger community.

“When we meet and interact as programme directors, I think it is easier to reach general decisions that apply to all. It’s bit of a shame for residents who perhaps may be on such units in which the programme director doesn’t manage to implement anything.” (IP16).

In a couple of the narratives, a former well-functioning work alliance had been terminated, which had negative outcomes for the change projects and sometimes resulted in the PD wanting to resign from their role.

### Having a vision and meaning

The PDs narratives expressed having a vision and meaning for the projects in terms of a conviction that the changes to be implemented were important and would have a positive impact on the quality of education. The meaningfulness of the change was expressed as being clear from the very beginning of the process of initiating the change. Examples of meanings experienced included increasing the independence of residents, or enhancing the good recruitment base of the hospital department/healthcare centre. The PD having a clear aim and meaning for the project served as an inner motivator, both in the short-term (for example, the PD working very intensely on the project for a limited time period) and in the long-term (such as wanting to continue in the PD role).

“I think that if you create good basic conditions, the Residents will progress in their development faster and will become self-sufficient and take more responsibility, be more analytical. And I think that is so rewarding to see.” (IP 7).

Participants’ vision and meaning also served as a basis for justification for the change, as well as a driving force used in contact with others, which helped create commitment in the organisation through a positive attitude towards the proposed changes.

“My role is to speak up so that it is available, it is important for their education in relation to colleagues, you can say (…). No one has made any objection to it. They understand that it is important that Residents get their theoretical education” (IP 5).

### Having a mandate for change

During the analysis it was noted that all participants referred to their own mandate in different, often implicit ways. For example, a mandate was mentioned in the sense of being empowered to initiate and implement changes in the organisation without asking for permission at every step. This was manifest in the observation that most changes were initiated by the PD with only a few exceptions.

Two sub-themes emerged from the theme of having a mandate that were connected to successful change implementation. The first was experiencing trust and power from superiors.

“And somehow, the health selection manager has delegated this to us/PDs/. And I think that helps us to be daring and feel that we want to take responsibility for the education and training of residents.”(IP 15).

Trust was often described as a fixed trait of each manager but could also be built by consciously and regularly anchoring and involving superiors. Some PDs described having experienced a lack of trust from superiors, which had resulted in reduced motivation or termination of the role as a PD.

The second sub-theme is that PDs reported that they felt the mandate was a result of their being experts in PGME. This included their knowledge about regulations for specialist training and how the training is carried out in departments and healthcare centres.

“It is quite easy to take up a mandate when you can refer to the fact that you do not actually meet the statutory requirements of the National Board of Health and Welfare.” (IP 12).

Another part of the expert knowledge leading to a mandate was participants’ clinical or managerial experience, which ultimately contributed to their informal power and mandate to make decisions regarding and run change projects.

### Involving colleagues and superiors

PDs initiated activities to create understanding, motivation towards and participation in the change. The participants’ narratives contained two sub-themes: anchoring and facilitation. Anchoring involved repeatedly performed activities with different target groups, such as gathering of views, workshops and conveying information. Special emphasis was placed on superiors at different levels.

“So, we have always gone through the managers, informing about what we want and have then requested the time from the management team. And I think that’s probably been an important and key factor for me the whole time. (…) And then I have often had discussions with the supervisors and the Residents. What are they thinking? How are they thinking? And what do they like, then we have… then I have also thought that it was easy to run the issue and raise it.” (IP 10).

Anchoring was the most pronounced and conscious strategy, primarily focused on the early steps in the implementation process. The narratives gave a picture of a long-term, time-consuming and sometimes frustrating work in which the PDs often had to deal with some resistance.

The sub-theme of facilitation was illustrated by stories of how PDs facilitated situations for others to act on the decided change. This included the provision of advice and practical administrative assistance. In changes related to workplace-based assessment, education activities aimed at the faculty were frequently used.

“We help them with programme items, how to structure it and produce something like this template, so you can use it as a course in the end. Yes, perhaps a little more around the practical aspects, how you do it. But then they have very quickly become self-reliant.” (IP 5).

While many PDs engaged in facilitation, it was rarely mentioned as an active, manifest strategy.

### Having a long‐term perspective

The PDs reported they had a long-term perspective on the change process, which manifested itself in two interconnected sub-themes - the approach to the change process (“having patience”) and how the change was practically implemented (“execution of the change as a step-by-step process”).

“It took quite a long time, it took a couple of years before I got through this and was then just chipping away, nagging the manager. (…) It is the straw that broke the camel’s back. A long-term work that allows you to get your wishes through. A lot of the time you won’t not get them through either the first, second or even third time but you keep at it and keep repeating something that you think should be done. And now it’s been established. Now it sits almost ingrained in the walls.” (IP 11).

The sub-theme of having patience was linked to the theme of having meaning and vision (presented above). The PDs expressed a confidence in the meaningfulness of the project, which gave them added patience when a project required extra time for implementation.

The sub-theme of change implementation as a step-by-step process gathered narratives on different change activities that were executed over longer period of time and an understanding that change takes time. Some participants described having a defined strategy prior to starting the implementation. However, the majority of PDs did not and focused on activities for the first phases of the change process (initiation and implementation).

Despite the understanding that change takes a long time, several stories emphasised that at some point the PD just had to initiate the change, despite resistance.

“And as we’ve talked about it for quite some time but we never really hit the mark because those who want to introduce it, were on parental leave or are on the verge of despair, so we then put our foot down and said: Now, we just have to run with it.” (IP 7).

## Discussion

This qualitative study describes different dimensions related to how PDs who had a prior positive impact on PGME implemented changes. We found similarities in how these PDs described and carried out the change initiatives. The similarities were manifest as five interrelated themes: belonging to a group, having a vision and meaning, having a mandate for change, involving colleagues and superiors, and having a long-term perspective. The themes included both behaviours (such as involving the faculty) and cognitive processes and aspects (such as regarding the change as meaningful).

Several themes identified in this study are similar to those of the change processes described by Kotter. [12] As suggested by Kotter’s model, participants focused on creating engagement within the organisation by involving colleagues and superiors, and by communicating at different organisational levels. The result also supports the importance of having strategic vision and working in a coalition. However, in comparison to other stages of the change process, the PDs appeared to give less attention to the final stages of change aimed at maintaining the change. This may be because the changes described had been recently implemented. However, a potential risk could be that the PDs focused on the early stages in implementation with the potential for negative implications for sustainability. As in Edmundson’s [16] work on how organizations learn, our study highlighted the importance of framing and setting a clear vision, as well as the importance of belonging to a team to establish psychological safety.

As earlier research has shown [23], the PDs in this study demonstrated limited awareness of the different steps in the change process and therefore might not plan the project with a clear strategy throughout the entire change process. One possible explanation for this is that change management support may have been absent, which are factors that have been pointed out as important in earlier studies. [21, 36] Another could be that the PDs are more effective at person-oriented than task-oriented behaviours, which make them more likely to focus on communicating activities. In accordance with an earlier study on successful change management [19], PDs in this study described mainly group- and systemic-focused behaviours (i.e. framing the change by communicating in the organisation and facilitating) as contrary to leader-centric behaviours focusing on a more “heroic” leadership role.

The concept of mandate is central to our study. The PDs described a mandate as something that has to be taken, not merely given. The point at several strategies to enhance the mandate, as referring to being an expert in PGME, working together with others, and deliberately involving superiors. One possible explanation might be the PDs’ relative lack of formal authority, in combination with conflict over available resources and focus on personal power sources and collaboration in order to successfully lead changes.

Our findings illuminate important aspects of successful change management in PGME. The study highlights the importance of the mandate and the degree to which successful change leadership in PGME is dependent on the conditions provided by next managerial level.

### Strengths and limitations

The present study explores successful change implementation with a focus on the role of the PD. The resulting themes complement existing theories by adding knowledge that having a mandate is more central to successful change management by PDs than suggested by general theories of change management. The primary limitation of our study is the small sample size, which limits our ability to generalise from the findings. The strategic inclusion of participants with different specialties, experiences and geographic regions in this study was to enhance transferability. Another possible limitation is the selection of participants. The research team wanted to understand change implementation from the perspective of PDs who successfully implemented change projects but lacked objective measures of success for the PDs’ projects. It is noteworthy that the findings of this study are congruent with earlier studies and the results may by transferable to PDs beyond our sample.

### Future research

Our findings help point the way forward for future research. Additional studies should determine whether the themes identified in this study also apply to another PD cohorts. As the context of a mandate was central to PDs leading change in this study sample, future research may investigate how a stronger mandate could be facilitated from a supervisor or organisational perspective.

### Implication for practice

This study offers a list of dimensions that should be taken into account at both an individual and an organisational level when planning and implementing a change within PGME. For the individual PD, the study shows that change should be based on a clear vision and is ideally performed in coalition with others. A long-term strategy should be planned, including involvement and anchoring of key persons in several steps. Our findings also suggest that leadership development of PDs could be beneficial in raising their awareness of their role as change agent and to further develop their competence for change management.

## Data Availability

The corresponding author, Hanna Wijk, can be contacted with queries relating to data. The datasets (interview transcripts) are not publicly available due to reasons of confidentiality.

## Abbreviations

PGME: Postgraduate medical education
PD: Programme director
